# Identification of Putative Bacterial Pathogens for Orofacial Granulomatosis Based on 16S rRNA Metagenomic Analysis

**DOI:** 10.1128/spectrum.02266-22

**Published:** 2023-05-25

**Authors:** Yuanyuan Wang, Hao Xu, Minghui Wei, Yuhong Wang, Wenzhe Wang, Jia Ju, Yuan Liu, Xinwen Wang

**Affiliations:** a Department of Oral Medicine, School of Stomatology, the Fourth Military Medical University, Xi’an, China; b Shaanxi Clinical Research Center for Oral Diseases, the National Clinical Research Center for Oral Disease of China, State Key Laboratory of Military Stomatology, Xi’an, China; c Shaanxi Key Laboratory of Brain Disorders & School of Basic Medical Sciences, Xi'an Medical University, Xi’an, China; d Department of Pharmacy, School of Stomatology, the Fourth Military Medical University, Xi’an, China; e Department of Oral Histology and Pathology, School of Stomatology, the Fourth Military Medical University, Xi’an, China; University of Guelph College of Biological Science

**Keywords:** apical periodontitis, granulomatous inflammation, orofacial granulomatosis, *Proteobacteria*, *Neisseria*

## Abstract

Orofacial granulomatosis (OFG) is a chronic inflammatory disease characterized by nontender swelling of the orofacial tissues, the underlying cause of which remains unknown. Our previous study demonstrated that tooth apical periodontitis (AP) is involved in the development of OFG. To characterize the AP bacterial signatures of OFG patients and identify possible pathogenic bacteria that cause OFG, the compositions of the AP microbiotas in OFG patients and controls were compared using 16S rRNA gene sequencing. Pure cultures of putative bacterial pathogens were established by growing bacteria as colonies followed by purification, identification, and enrichment and then were injected into animal models to determine the causative bacteria contributing to OFG. A specific AP microbiota signature in the OFG patients was shown, characterized by the predominance of phyla *Firmicutes* and *Proteobacteria*, notably members of the genera Streptococcus, *Lactobacillus*, and *Neisseria*, were found. Streptococcus spp., Lactobacillus casei, Neisseria subflava, Veillonella parvula, and *Actinomyces* spp. from OFG patients were isolated and successfully cultured *in vitro* and then injected into mice. Ultimately, footpad injection with *N. subflava* elicited granulomatous inflammation.

**IMPORTANCE** Infectious agents have long been considered to play a role in the initiation of OFG; however, a direct causal relationship between microbes and OFG has not yet been established. In this study, a unique AP microbiota signature was identified in OFG patients. Moreover, we successfully isolated candidate bacteria from AP lesions of OFG patients and assessed their pathogenicity in laboratory mice. Findings from this study may help provide in-depth insights into the role of microbes in OFG development, providing the basis for targeted therapeutic approaches for OFG.

## INTRODUCTION

Orofacial granulomatosis (OFG) is a chronic inflammatory disease characterized by nontender swelling of the orofacial tissues. It might be idiopathic, localized only in the skin and subcutaneous tissue of the oral cavity, and might also be present in several diseases, such as Crohn’s disease (CD), sarcoidosis, and Melkersson-Rosenthal syndrome (1, 2).

Infectious agents have long been considered to play a role in the initiation of OFG. Although antibodies against mycobacterial stress protein (3) and the spirochete Borrelia burgdorferi (4) were found in some patients with OFG, the results were not confirmed by further studies (5). With the development of high-throughput sequencing technology of 16S rRNA, the microbiome composition of saliva from OFG patients was explored, but the results were inconsistent in different studies (6, 7). So far, a direct causal relationship between microbe and OFG has not yet been established, and the underlying cause of OFG remains to be elucidated.

In our previous study we found that 71.5% of the OFG patients had chronic tooth apical periodontitis (AP) (8), and they showed a marked response after receiving dental treatment, highlighting the involvement of AP in the pathophysiology of OFG. Therefore, the purpose of this study was to finely characterize the microbiotas present in AP of OFG patients by comparing them with those in AP patients not affected with OFG using a 16S rRNA metagenomic approach. Moreover, we isolated and cultured the candidate bacteria directly from AP of OFG patients and demonstrated the pathogenicity of the candidate bacteria using experimental animal models.

## RESULTS

### Composition of AP microbiotas in OFG patients.

**FIG 1:**
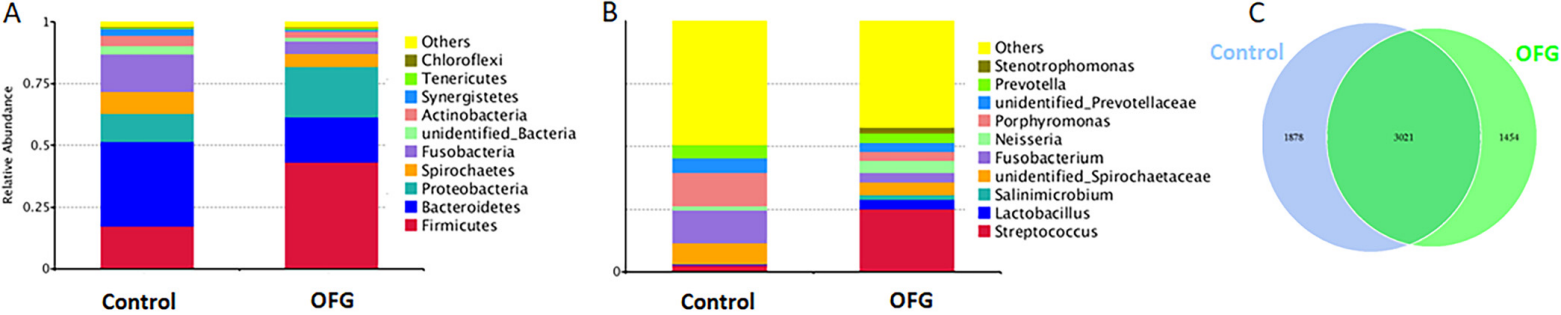
Composition of the AP microbiotas in OFG patients and control individuals. (A) Relative abundance of bacteria at the phylum level in the OFG and control groups. (B) Relative abundance of bacteria at the genus level in the OFG and control groups. (C) Venn diagram showing the unique and shared OTUs between the control and OFG samples.

The number of final clean reads per sample ranged from 50,416 to 127,314. Sequences with more than 97% similarity were considered one operational taxonomic unit (OTU). On average, 4,475 OTUs in the OFG group and 4,899 OTUs in the control group were detected, with 3,021 OTUs in common (Fig. 1). They were assigned to the phylum, class, order, family, and genus taxonomic levels. In total, 6,282 OTUs were successfully annotated, which were used to generate the taxonomy tables and rarefaction curves, calculate species richness and diversity, and cluster samples with principal-coordinate analysis (PCoA).

The phyla *Bacteroidetes*, *Firmicutes*, *Proteobacteria*, *Fusobacteria*, and *Spirochaetes* accounted for the majority of the sequences. In the control group, *Bacteroidetes* was the most prevalent phylum, followed by *Firmicutes*, *Fusobacteria*, and *Proteobacteria*, whereas the OFG group exhibited a specific AP microbiota signature. The phylum *Firmicutes* had the highest relative abundance, followed by *Proteobacteria* and *Bacteroidetes* (Fig. 1A). At the genus level, the OFG group had a greater abundance of Streptococcus (12-fold increase), *Lactobacillus*, *Neisseria*, *Salinimicrobium*, and *Stenotrophomonas* than the control group. *Porphyromonas*, *Fusobacterium*, an unidentified *Spirochaetaceae*, and *Prevotella* spp. accounted for the majority of the bacterial community in the control group (Fig. 1B).

We also analyzed the microbial community in the OFG biopsy tissue. Due to poor DNA quality, three samples from paraffin-embedded biopsy specimen were excluded. Ultimately, 2,483 OTUs in six OFG biopsy samples were detected. Figure S1 in the supplemental material shows the composition of the microbial communities among the six biopsy samples. *Proteobacteria* (14.9% to 58.4%) was the most abundant phylum, followed by *Firmicutes* (19.5 to 37.5%) and *Bacteroidetes* (4.5% to 21.0%). The communities in the six samples, at the genus level, are shown in Table S1. *Lactobacillus*, Streptococcus, and Pseudomonas were prevalent across all the samples.

### Diversity of AP microbiotas in OFG patients.

**FIG 2:**
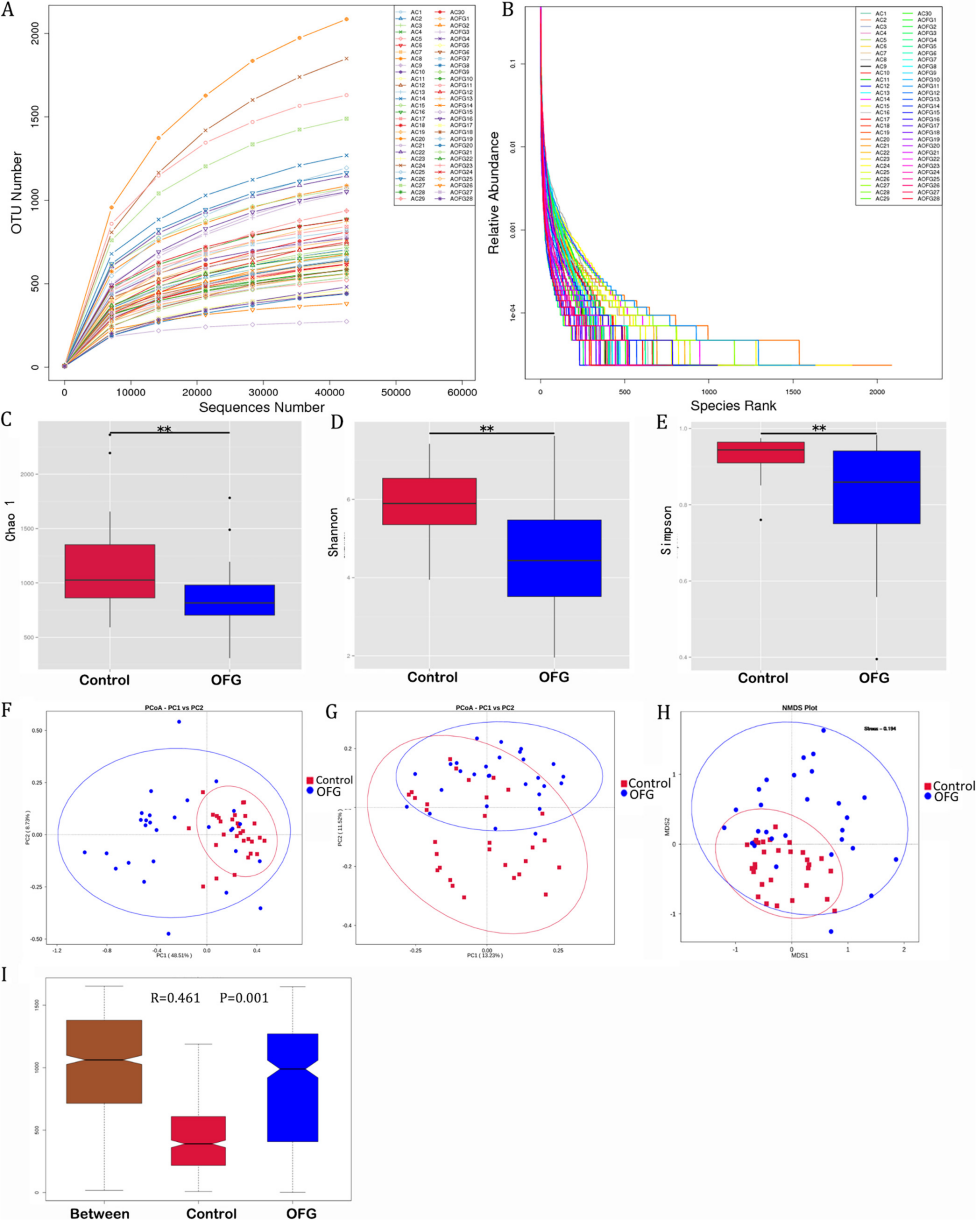
Alpha diversity and beta diversity analysis of AP microbiotas in the OFG group and the control group. (A) Alpha diversity rarefaction curve. (B) Rank abundance curves. (C to E) Chao 1 (C) Shannon (D), and Simpson (E) indices were calculated based on OTU relative abundance. (F) Clusters formed by PCoA based on weighted UniFrac distance matrix. Each dot represents a sample. (G) Clusters formed by PCoA based on unweighted UniFrac distance matrix. (H) Clusters formed by NMDS analysis. (I) ANOSIM showed a significant difference between the OFG and control group (*R* = 0.461, *P = *0.001). **, 0.001 < *P* < 0.01.

Bacterial richness and diversity were assessed by the Shannon, Chao 1, and Simpson indices. The results showed a significantly lower richness and α-diversity in the OFG group compared to that in the control (*P < *0.01) (Fig. 2). The results of the PCoA based on weighted UniFrac distance matrix and unweighted UniFrac distance matrix, nonmetric multidimensional scaling (NMDS) analysis, and analysis of similarity (ANOSIM) showed that the AP bacterial community in the OFG group was significantly different from that in the control group; they tended to form separate clusters and could be distinguished from each other. The OFG samples appeared more dispersive than the control samples (Fig. 2).

**FIG 3:**
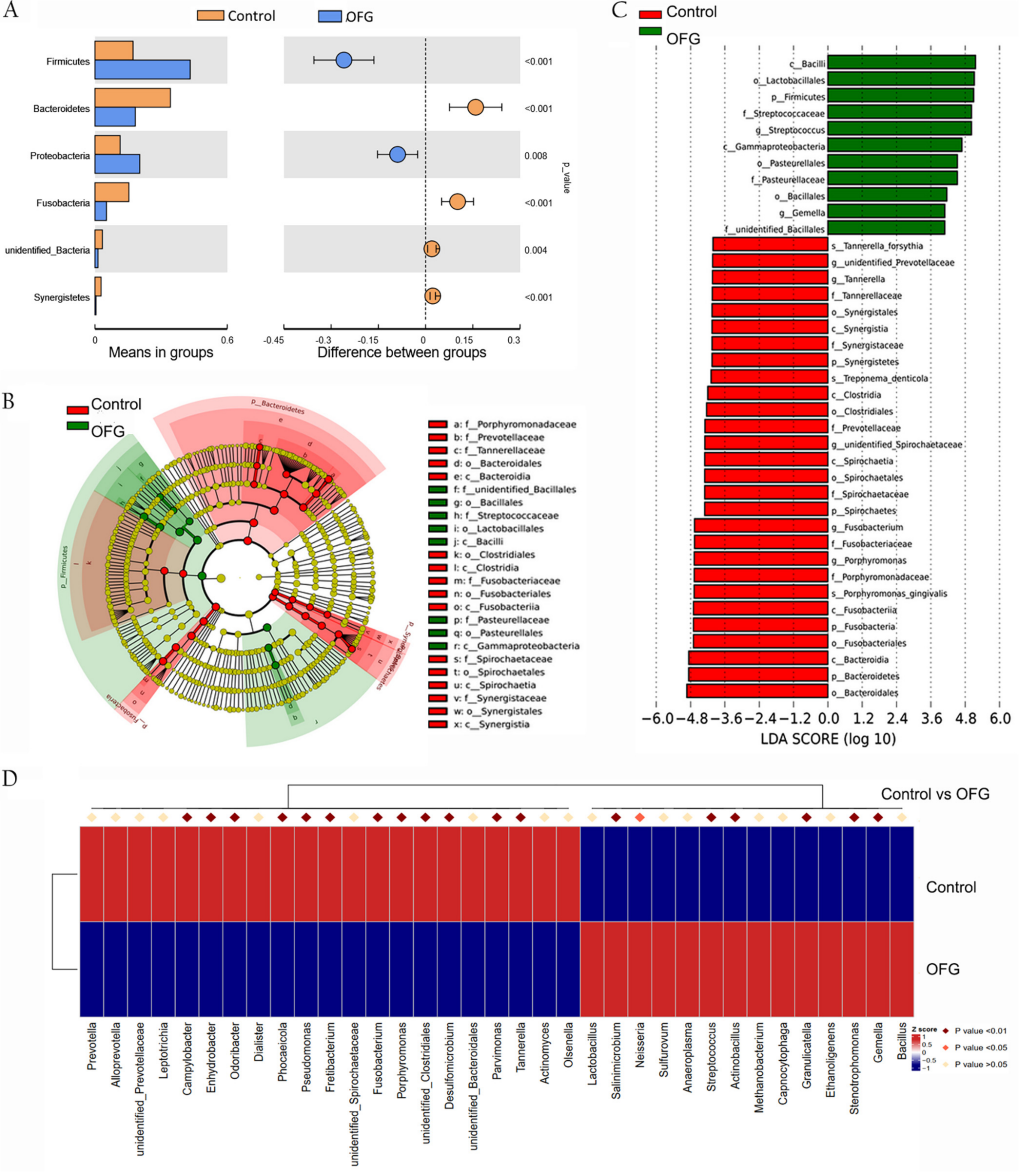
Differentially abundant taxa. (A) Phyla that were significantly more abundant in the AP bacterial communities of OFG patients and control individuals. (B) Taxa (highlighted by small circles and by shading) showing different abundance values in the two groups. (C) Taxa that were significantly more abundant in the two groups, as determined by LDA effect size analysis (LDA score ≥ 4). (D) The 35 most abundant genera from the control and OFG microbiomes are presented.

The differentially abundant class, order, family, and genus identifications between the two groups are shown in Fig. 3. *t* test results showed that the phyla *Firmicutes* and *Proteobacteria* were significantly overrepresented in the OFG group (*P < *0.001 and *P = *0.008, respectively) compared to their representation in the control group (Fig. 3A). The cladogram demonstrates the taxa with different abundances in the two groups (Fig. 3B). Linear discriminant analysis (LDA) effect size (LEfSe) was used to predict biomarker taxa in each group, and the results showed that at the class level, *Bacilli* and *Gammaproteobacteria* were significantly more enriched in the OFG group. In contrast, *Bacteroidia*, *Fusobacteriia*, *Spirochaetia*, *Clostridia*, and *Synergistia* were significantly overabundant in the control group (Fig. 3C).

### AP microbiotas in the OFG patients and control individuals exhibited different enrichment in metabolic pathways.

In this study, there were considerable AP microbiota-associated KEGG gene function changes in OFG patients. At KEGG level 2, we identified 31 different KEGG pathways that were significantly different between the OFG group and the control group (Fig. S2). The top six pathways significantly enriched in the OFG group were membrane transport (*P < *0.001), signal transduction (*P < *0.001), xenobiotics biodegradation/metabolism (*P < *0.001), carbohydrate metabolism (*P = *0.006), amino acid metabolism (*P = *0.045), and lipid metabolism (*P = *0.001). The top six pathways significantly enriched in the control group were translation (*P < *0.001), replication/repair (*P = *0.005), metabolism of cofactors/vitamins (*P < *0.001), nucleotide metabolism (*P < *0.001), transport/catabolism (*P < *0.001), and energy metabolism (*P = *0.003).

### Acquisition of candidate bacteria from OFG patients.

**FIG 4:**
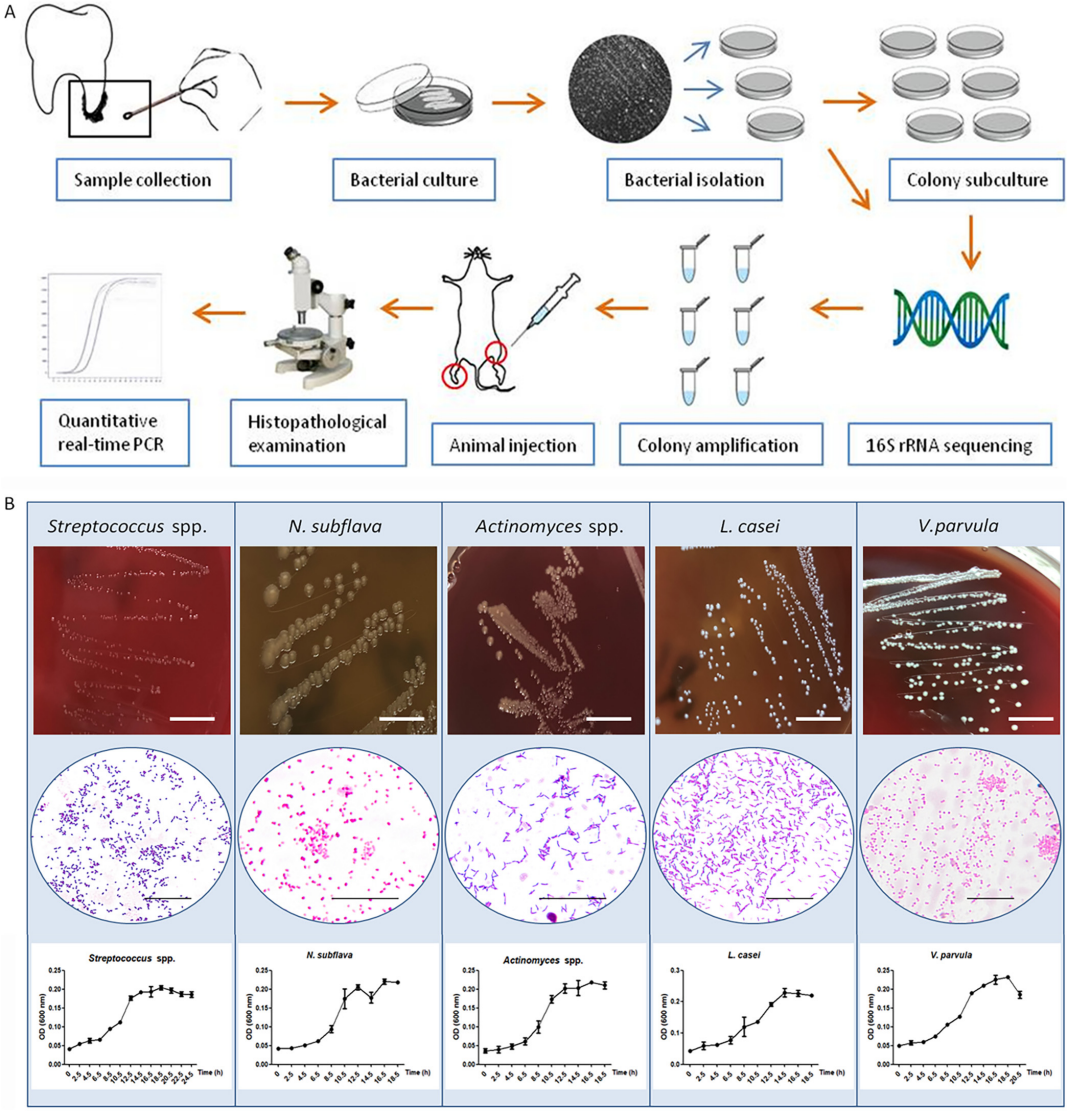
Bacterial isolation and animal experiments. (A) Overall workflow for bacterial isolation, culture, and animal experiments. (B) Gram staining and growth analysis of Streptococcus spp., *N. subflava*, *Actinomyces* spp., *L. casei*, and *V. parvula.* White bars, 1 cm; black bars, 1,000 μm.

As shown in Fig. 4A, bacterial samples were obtained from the root surfaces of the teeth from three OFG patients (one female age 45, one female age 53, and one male age 53), and were initially cultured under different conditions as described in Materials and Methods. All colonies with unique color or morphology and many colonies with similar color or morphology incubated under different conditions were collected for preliminary identification by 16S rRNA sequencing. Of the 30 isolates examined, Streptococcus spp. had the highest percentage of occurrence at 100%, followed by *Neisseria* spp. at 85.7%, *Actinomyces* spp. at 71.4%, Haemophilus spp. at 71.4%, *Veillonella* spp. at 71.4%, *Romboutsia* spp. at 64.3%, *Blautia* spp. at 57.1%, Escherichia spp. at 57.1%, *Faecalibacterium* spp. at 57.1% and *Lactobacillus* spp. at 42.9% (Table S2).

The isolates were subcultured to ensure purity, and then they underwent several *in vitro* passages. After the sequences were identified and compared to known sequences using a BLAST search in GenBank, pure cultures of Streptococcus spp., Neisseria subflava, Lactobacillus casei, Veillonella parvula, and *Actinomyces* spp. were established, and the average nucleotide identity (ANI) values were 99.54%, 99.22%, 99.85%, 99.67%, and 99.49%, respectively. For Streptococcus spp. and *Actinomyces* spp., definitive identification to the species level based on sequencing was difficult, although the sequencing results showed closest matches to members of Streptococcus and *Actinomyces* when compared to sequences in GenBank, so Streptococcus spp. and *Actinomyces* spp. were considered presumptive identifications.

As expected, Gram staining indicated that *N. subflava* and *V. parvula* were Gram negative and Streptococcus spp., *L. casei*, and *Actinomyces* were Gram positive (Fig. 4B). The growth of the putative bacteria (Fig. 4B) showed a slight increase over time under different conditions (Streptococcus spp., *L. casei*, and *V. parvula* under anaerobic conditions, *N. subflava* and *Actinomyces* under microaerobic conditions). Culture details are available in in the supplemental material.

### Effects of candidate bacterial infections in mice.

**FIG 5:**
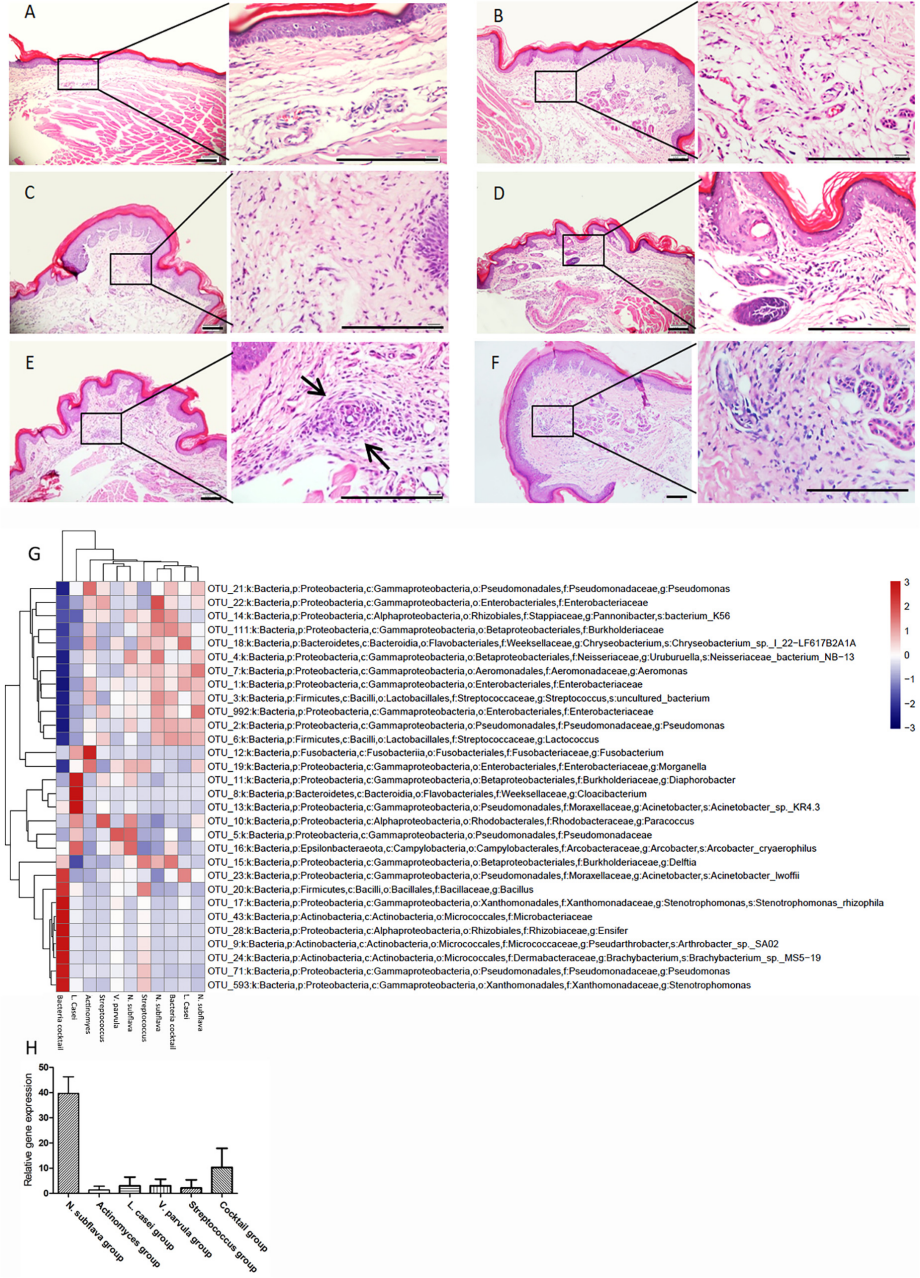
Photomicrograph of hematoxylin-and-eosin-stained tissues of the mice injected with different candidate bacteria. Histopathology of footpads injected with (A) Streptococcus spp., (B) *L. casei*, (C) *V. parvula*, (D) *Actinomyces* spp., or (F) a bacterial cocktail revealed a relatively normal appearance of subcutaneous tissue or sparse inflammatory cell infiltration. (E) Dermal inflammatory infiltration composed of epithelioid cells (arrows) was observed in footpads of mice injected with *N. subflava*. Bar, 100 μm. (G) Relative abundance heat map of the 30 OTUs identified as differentially abundant in the tissue samples. (H) *N. subflava* distribution among different samples analyzed by qPCR.

After 20 days, the mice injected with Streptococcus spp., *L. casei*, *V. parvula*, *Actinomyces*, and a bacterial cocktail showed a relatively normal histomorphology, although a few demonstrated sparse inflammatory cell infiltration (Fig. 5). A granulomatous reaction was observed in seven of 10 mice infected with *N. subflava* in the footpads (Fig. 5E). Histological examination of ear and leg biopsy specimens showed no obvious infiltration of inflammatory cells (Fig. S3). The relative-abundance heat map of the OTUs identified as differentially abundant in the tissue samples is shown in Fig. 5G. The quantitative PCR (qPCR) targeting the gene encoding aspartate semialdehyde dehydrogenase (9) revealed that the positive samples contained four to seven times as many *N. subflava* as the other groups (Fig. 5H).

## DISCUSSION

The mouth connects the body to the external environment and represents one of the most biologically complex regions. The oral microbiota composition is readily changeable by oral hygiene, diet, site, temperature, various oral diseases, and other life events (10). The complexity of the oral microbiota poses a significant challenge to the analysis and interpretation of the alteration in the oral bacterial community, which partly underlies previous inconsistent results regarding the salivary microbiota associated with OFG (6, 7). AP is a common inflammatory disorder of the dental periradicular tissue, which results from infections of the root canal of the endodontically involved teeth (11, 12). In this study, we collected microbial samples from the tooth root surface with AP, avoiding contamination from the oral cavity and reducing the effect of influencing factors as much as possible. The results showed that the composition of AP microbiotas in the control group was largely similar to that found in previous studies (13–16), indicating that the bacterial community in AP was relatively stable. Together with the fact that AP is a high-risk factor for OFG (8), these findings indicate that the AP microbiota provided an ideal target for OFG research and ensured the reliability of the analysis.

Our results demonstrated that the richness and diversity of the AP bacterial communities in OFG patients were significantly decreased compared to that of the control group (4,475 OTUs versus 4,899 OTUs). The decreased diversity was consistent with the observation in CD, which is another granulomatous inflammation (GI) disease that is closely related to OFG in the Caucasian population (17). However, an increase in *Proteobacteria* was observed in patients with OFG, as was reported in gut mucosa-associated microbiotas in CD patients (18, 19). The present study demonstrated that AP in OFG patients consisted of a microbiota that differed significantly from that in the control group. The phyla *Proteobacteria* and *Firmicutes* were more abundant than the phyla *Bacteroidetes* and *Fusobacteria*, which are usually the most abundant in AP microbiota (14, 15). This result was further confirmed in OFG biopsy tissue.

*Proteobacteria* is one of the most abundant phyla within the human body and is divided into six classes based on the phylogenetic analysis of the 16S rRNA (*Alphaproteobacteria*, *Betaproteobacteria*, *Gammaproteobacteria*, Deltaproteobacteria, *Epsilonproteobacteria*, and “*Candidatus* Zetaproteobacteria”) (20). There are many *Gammaproteobacteria* members, including Escherichia coli (21), Francisella tularensis (22), *Yersinia* (23), Salmonella enterica serovar Typhi (24), and *Burkholderia* (25), that have been reported to be associated with the development of a range of GI diseases. Our study demonstrated that *Gammaproteobacteria* was significantly correlated with OFG. As a member of *Gammaproteobacteria* (https://www.arb-silva.de/), *Neisseria* was found to be one of the top enriched taxa in both AP and biopsy tissues of OFG patients, highlighting its important role in OFG pathogenesis.

*Neisseria* spp. are generally restricted to humans (26), and most *Neisseria* spp. are regarded as components of the host normal microbiota (27). However, increasing evidence suggests that these “harmless inhabitants” are capable of producing infection in different parts of the body (27–29). In this study, we isolated *N. subflava* from the infected root surface of OFG patients with AP and injected the clinical isolate into an animal model. GI responses were observed in the footpads of mice, and *N. subflava* could be detected in the pathological lesions in these mice, which further suggested the pathogenicity of *N. subflava* in OFG. Interestingly, no GI formed in the legs and ears injected with *N. subflava* or the bacterial cocktail or in footpads injected with the bacterial cocktail, suggesting that the anatomical site and the number of bacteria may have a certain influence on the formation of GI.

The role of Streptococcus in OFG pathogenesis was investigated in previous studies, but the results were unclear, as an increase in the abundance of Streptococcus in the salivary microbiotas of OFG patients was reported (6) and a reduction in the abundance was also reported (7). In our study, *Firmicutes* was the most abundant phylum in the OFG group, and Streptococcus contributed the most to the high proportions of *Firmicutes*. Although we did not observe a GI reaction in animal models, in the study by Sartor et al. (30), the injection of cell wall fragment of Streptococcus into the small intestine and cecum was shown to induce granulomatous enterocolitis in rats, suggesting that its role still should be investigated further.

Previous microbiological and molecular studies showed that fermentation end products of various bacteria in deep caries are closely related to clinical pain or hypersensitivity (31). The carious teeth with high *Lactobacillus* counts are not sensitive to stimuli (32, 33), as the organic acids produced by *Lactobacillus* not only fail to excite the intratubular Aδ nerves but also suppress nerve impulses elicited by other stimuli (34). In this study, we found that *Lactobacillus* was consistently enriched in AP samples and biopsy tissues of OFG patients. This might partly explain why the AP in OFG patients was asymptomatic, even when several teeth were affected simultaneously (8).

AP is a chronic infection of endodontic origin; energy metabolism, especially carbohydrate metabolism, is essential for the development of AP. In this study, the predicted KEGG pathways significantly enriched in the OFG group were carbohydrate and amino acid metabolism, which are consistent with existing literature (35, 36). Interestingly, through function prediction, we found that membrane transport, signal transduction, and xenobiotic biodegradation pathways were enriched in the OFG group, which suggested that the AP microbiotas in patients with OFG may have fundamental influences on the communication with the host cells. Combining the metabolite and microbiota analyses may help to address questions related to overall changes during OFG development and provide new insights into the disease pathology.

There are several limitations to our study. First, only five potential bacterial species were evaluated in the animal model. Recently, microbial dysbiosis in the commensal community has received greater attention, and different bacteria might play different roles in disease progression, so there is still a need to clarify whether other bacteria contribute to the development of OFG individually or jointly. Second, we injected the clinical isolates into mouse footpads to determine the causative bacteria contributing to OFG. As OFG is a complex pathological process involving multiple factors, it is hard to simulate the pathological microenvironment completely in mice, and so the virulence of the putative bacteria as OFG pathogens still needs to be further confirmed by clinical examination.

In conclusion, this study demonstrated a specific AP microbiota signature in patients with OFG. We successfully isolated candidate bacteria from AP lesions of OFG patients, assessed their pathogenicity in laboratory mice, and obtained results suggesting that *N. subflava* might be an important pathogen for OFG. Findings from this study may help provide in-depth insights into the role of this microbe in OFG development that could impact the management of OFG in the future.

## MATERIALS AND METHODS

### Ethics statement.

The study was approved by the Ethics Committee of the School of Stomatology, the Fourth Military Medical University (FMMU) (approval IRB-REV2020039), and was conducted according to the code of ethics of the World Medical Association (Declaration of Helsinki). Animal experiments were approved by the Animal Care and Use Committee of the institute (no. 20200903) and complied with the ARRIVE checklist (ARRIVE 2.0). Male wild-type (WT) BALB/c mice (6 to 8 weeks old) were bred in a specific-pathogen-free environment at the Animal Care Center of the School of Stomatology, FMMU, and were used for the experiments.

### Participants.

Twenty-eight OFG patients with AP (22 females; average age, 45.3 ± 10.2 years) were included in the study group. The control group consisted of 30 patients with only AP (20 females; average age, 51.7 ± 16.2 years). All participants signed an informed-consent form before they participated in the study. A diagnosis of OFG was determined based on the currently accepted criteria (37–39). Complete medical history and panoramic/periapical radiograms of each patient were assessed. Participants were selected by applying the following exclusion criteria: hematologic diseases, uncontrolled diabetes, disease of the immune system, or use of topical/systemic antibiotics or immune agents within 1 month. The tooth samples were selected if they were clinically diagnosed as having AP and required extraction because they were judged unrestorable.

### DNA extraction and 16S rRNA sequencing.

Before tooth extraction, participants rinsed their mouths with 10 mL 0.9% sterile physiological saline solution for 1 min. Cotton rolls were placed into vestibular groove to avoid contamination with saliva. Immediately after extraction, microbial samples were taken from the apical part of the root with a sterile flocked swab (MEDICO, China); care was taken to avoid saliva contamination. The samples were frozen at −20°C.

DNA was extracted using the cetyltrimethylammonium bromide (CTAB)/SDS method. The V3-V4 region of the bacterial 16S rRNA genes was amplified using a specific primer (338F-806R). The details of gene sequencing and taxonomic analysis are available in the supplemental material. The findings were further confirmed in the OFG biopsy tissue (see the supplemental material).

### Isolation of putative bacterial pathogens and animal experiments.

The apical content harvested from the extracted teeth of OFG patients was used for isolation of putative bacterial pathogens. To determine whether the putative bacterial pathogens could cause pathological changes similar to those observed in OFG patients, we injected the male WT BALB/c mice with these organisms. Further details are available in the supplemental material.

### Statistical analysis.

For the 16S rRNA gene sequencing data analysis, the Wilcoxon and *t* tests were used to evaluate the differences in the bacterial populations between the OFG group and the control group. Within-community diversity (alpha diversity) was calculated by Chao richness, Shannon index of species, and Simpson index. To assess the differences in the bacterial community between the two groups, principal-component analysis (PCA) based on the OTUs, PCoA based on UniFrac, and NMDS were performed. ANOSIM was conducted using the distance matrix to obtain statistical confidence for the sample grouping observed by PCoA. LEfSe software was used to analyze differentially abundant taxa; the filter value of the LDA score was 4 by default (*P < *0.05).

### Data availability.

Data sets are publicly available at NCBI under the accession number PRJNA954326.
