# Supplementary material for: Identification of Putative Bacterial Pathogens for Orofacial Granulomatosis Based on 16S rRNA Metagenomic Analysis
Source: Microbiol Spectr. 2023 May 25;11(3):e02266-22. doi: 10.1128/spectrum.02266-22 (PMC10269498; doi:10.1128/spectrum.02266-22)
Supplement: Supplemental file 1 — Supplemental material. Download spectrum.02266-22-s0001.pdf, PDF file, 0.8 MB [file spectrum.02266-22-s0001.pdf]

DNA extraction from biopsy tissue and sequencing

We analyzed formalin-fixed and paraffin-embedded tissue from OFG patients, which were taken from maxillofacial soft tissue at the site of the disease. Five slices of tissue samples were used for total genomic DNA extraction, and a specific primer (515F-806R) was used for the amplification of the V4 region of the bacterial 16S rRNA gene. After the preparation and generation of the 16S rDNA library, the quality of the library was assessed using the Qubit@ 2.0 Fluorometer (Thermo Scientific) and Agilent Bioanalyzer 2100 system. The library was sequenced using the Illuminal HiSeq2500 platform, and paired-end reads of 250-bp were generated.

Taxonomic analysis

Sequence analysis was performed using the Uparse software (Uparse v 7.0.1001). Sequences with >97% similarity were assigned to the same OTUs, and the Silva Database was used to annotate taxonomic information based on the RDP classifier algorithm. The information on the abundance of the OTUs was normalized using a standard for the sequence number corresponding to the sample with the lease sequences.

The alpha and beta diversity based on the output of the normalized data, were used to compare the differences of AP microbiota in OFG patients and control individuals. Unweighted UniFrac was used to measure the distance between two bacterial communities by calculating the fraction of the branch length in a phylogenetic tree. Weighted UniFrac was used to measure the distance between two bacterial communities by comparing the genetic diversity within each community to the total genetic diversity of the communities combined.

Functional predictions

The Tax4Fun software package was used to predict the functional capabilities of bacterial communities based on the SILVA-labeled OUT abundances. The SILVA-based 16S rRNA datasets were transformed to a taxonomic profile of the prokaryotic KEGG organisms, which were then normalized by the 16S rRNA copy number. The normalized taxonomic abundances were used to predict the functional profile of the bacterial community.

Isolation and culture of putative bacterial pathogens

The apical content was harvested from the extracted tooth of OFG patients using a sterile swab, and was placed in a sterile tube to avoid contamination by microorganism. The bacterial culture of the inflammatory content was performed on various media plates under different conditions.

Plates of brain heart infusion (BHI) agar and BHI agar with 5% sheep blood were incubated at 35℃ under anaerobic (80% N_2, 10% H_2, 10% CO_2) and microaerobic (5% O_2, 12% CO_2) conditions at the same time. Preliminary identification of bacterial isolates that were different in appearance was performed using 16S rRNA based on a metagenomic approach as described earlier.

Individual colonies were then subcultured. When growth was clearly visible, these colonies were harvested and cultured further to ensure purity. The 16S rRNA gene of each kind of colony was sequenced using the MicroSeq Full Gene 16S rRNA Bacterial Isolation Sequencing Kit (Applied Biosystems). The complete genome was used to identify the species in each colony. The pure cultures of *Streptococcus* spp., *L. casei, N. subflava, V. parvula* and *Actinomyces* spp. identified through 16S rRNA sequencing served as the source of the bacteria used in the following study.

Monolayer of *Streptococcus* spp., *L. casei, N. subflava, V. parvula* and *Actinomyces* spp. were prepared in culture plates. After fixing, the monolayer was washed with PBS, and stained with Gram. The plate was then examined microscopically under bright field microscope (Nikon Eclipse TS100). To assess bacterial growth, a 1ml sample of the stationary phase culture of the overnight growth (diluted to 1×10^{8}CFU/ml) was transferred to 10ml of BHI medium. All test tubes were incubated at 35℃ (*Streptococcus* spp., *L. casei,* and *V. parvula,* 80% N_2, 10% H_2, 10% CO_2, *N. subflava* and *Actinomyces* spp., 5% O_2, 12% CO_2), and standard growth curve fits were prepared by plotting absorbance readings at 600nm (OD_{600nm}).

Animal experiments

After the bacteria were harvested by centrifugation, the bacterial pellets were diluted to 1×10^{7} CFU/ml. For each bacteria species, ten mice were injected into the footpads, ten mice were injected into the ears, and ten were injected into the hind legs. The injection dose was 0.5 mL of bacteria suspension. At the same time, bacteria cocktail (*Streptococcus* spp./*N. subflava*/*L. casei*/*V. parvula*/*Actinomyces* spp.=*1:1:1:1:1*) was injected at the same dose.

After 20 days, the mice were sacrificed. Ears, pads and legs were cut sagittal sections, half of the tissue was used for DNA extraction, while the other half of the tissue was fixed with 10% neutral-buffered formalin and embedded in paraffin. Sections (5μm thick) were prepared and mounted on glass slides, then were stained with H&E. DNA was extracted from the tissue, and the amount of the candidate bacteria was determined by probe-based quantitative real-time PCR

(q-PCR).
