# Supplementary material for: Identification of Putative Bacterial Pathogens for Orofacial Granulomatosis Based on 16S rRNA Metagenomic Analysis
Source: Microbiol Spectr. 2023 May 25;11(3):e02266-22. doi: 10.1128/spectrum.02266-22 (PMC10269498; doi:10.1128/spectrum.02266-22)
Supplement: Supplemental file 2 — Supplemental material. Download spectrum.02266-22-s0002.pdf, PDF file, 1.5 MB [file spectrum.02266-22-s0002.pdf]

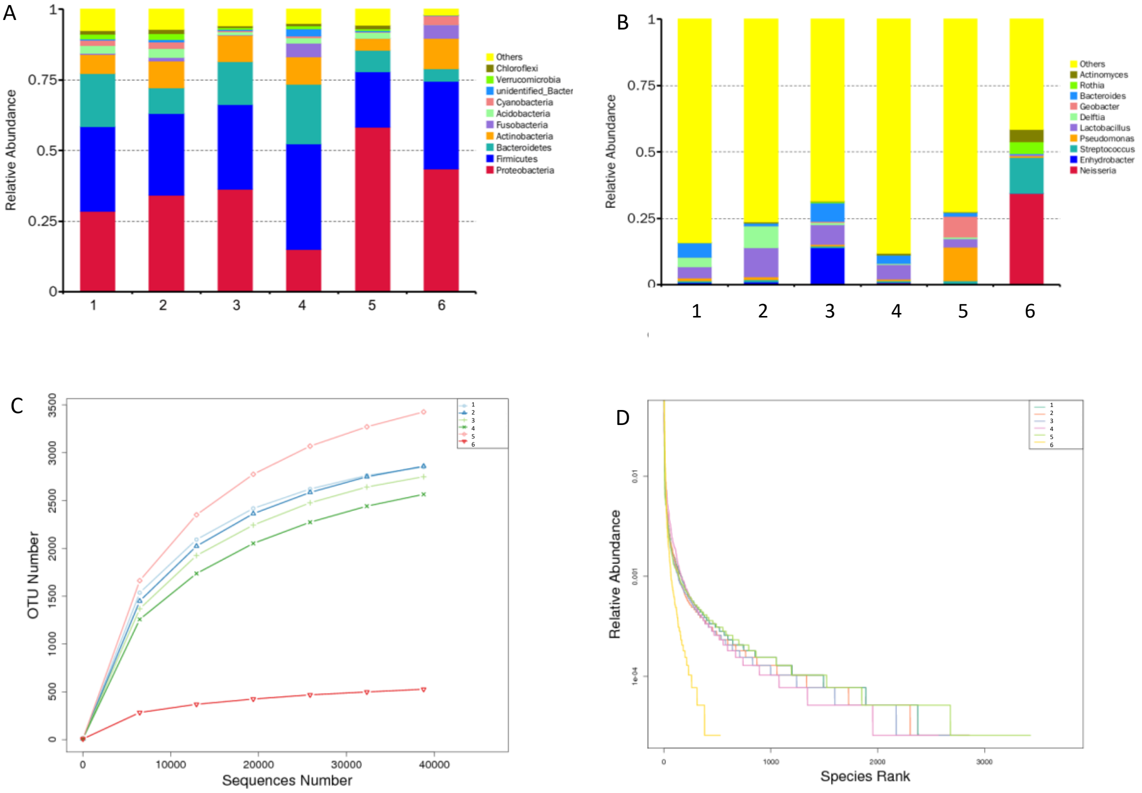

Appendix Figure 1. Bacterial profiles of the OFG biopsy tissues. (A) Top 10 different bacterial phyla in the OFG biopsy tissues. (B) Top 10 different bacterial genera in the OFG biopsy tissues. (C) Alpha diversity rarefaction curve. (D) Rank abundance curves.

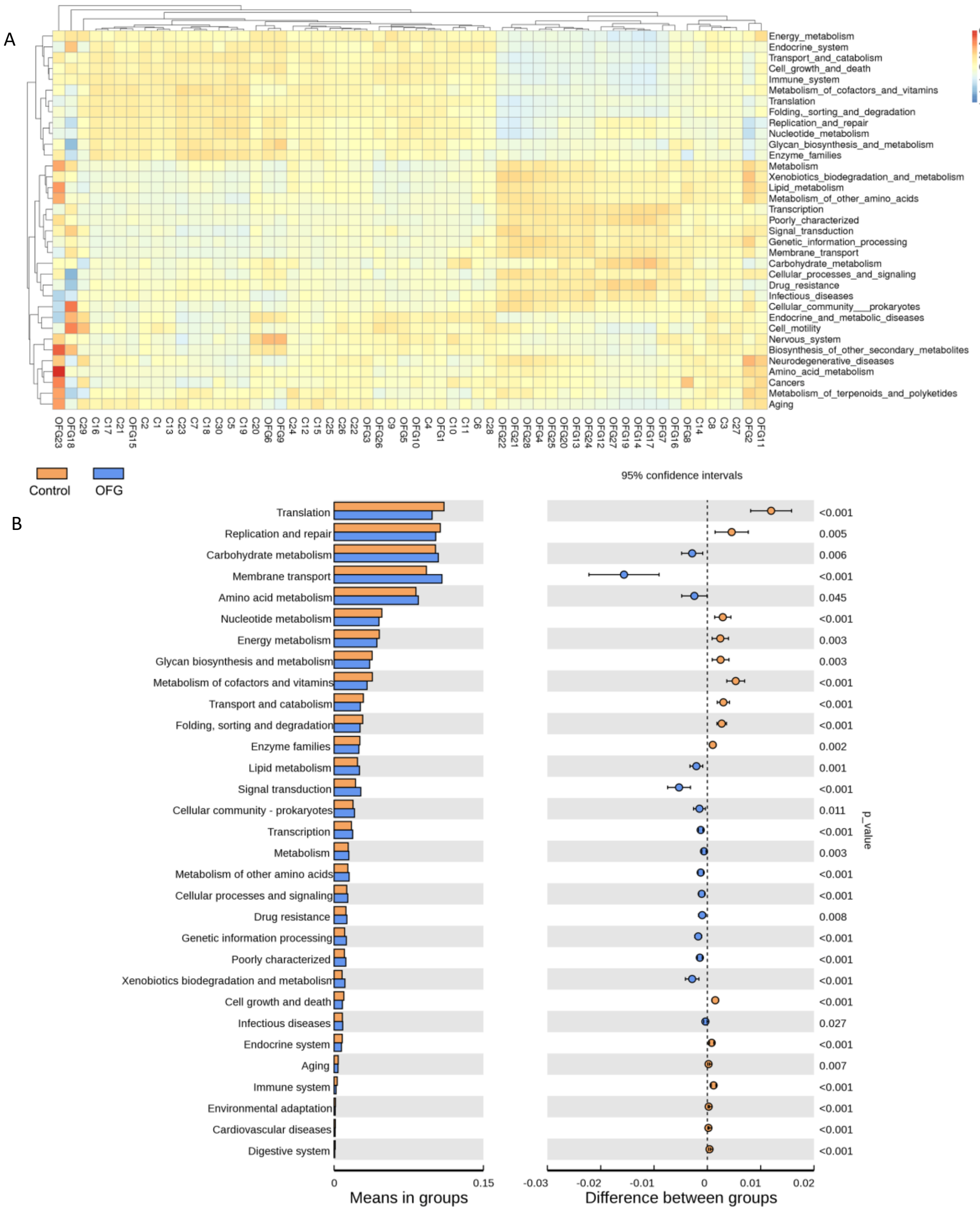

Appendix Figure 2**.** OFG patients and control individuals exhibited differential enrichment in AP microbial pathways. (A) Heat map for KEGG enrichment results. Metabolites are shown on the row in the format, sample IDs are shown on the column. (B) The significantly different microbial pathways between the two groups. Relative enrichment fold change is shown on the x axis, and the name of pathway is shown on the y axis. AP, apical periodontitis; OFG, orofacial granulomatosis.

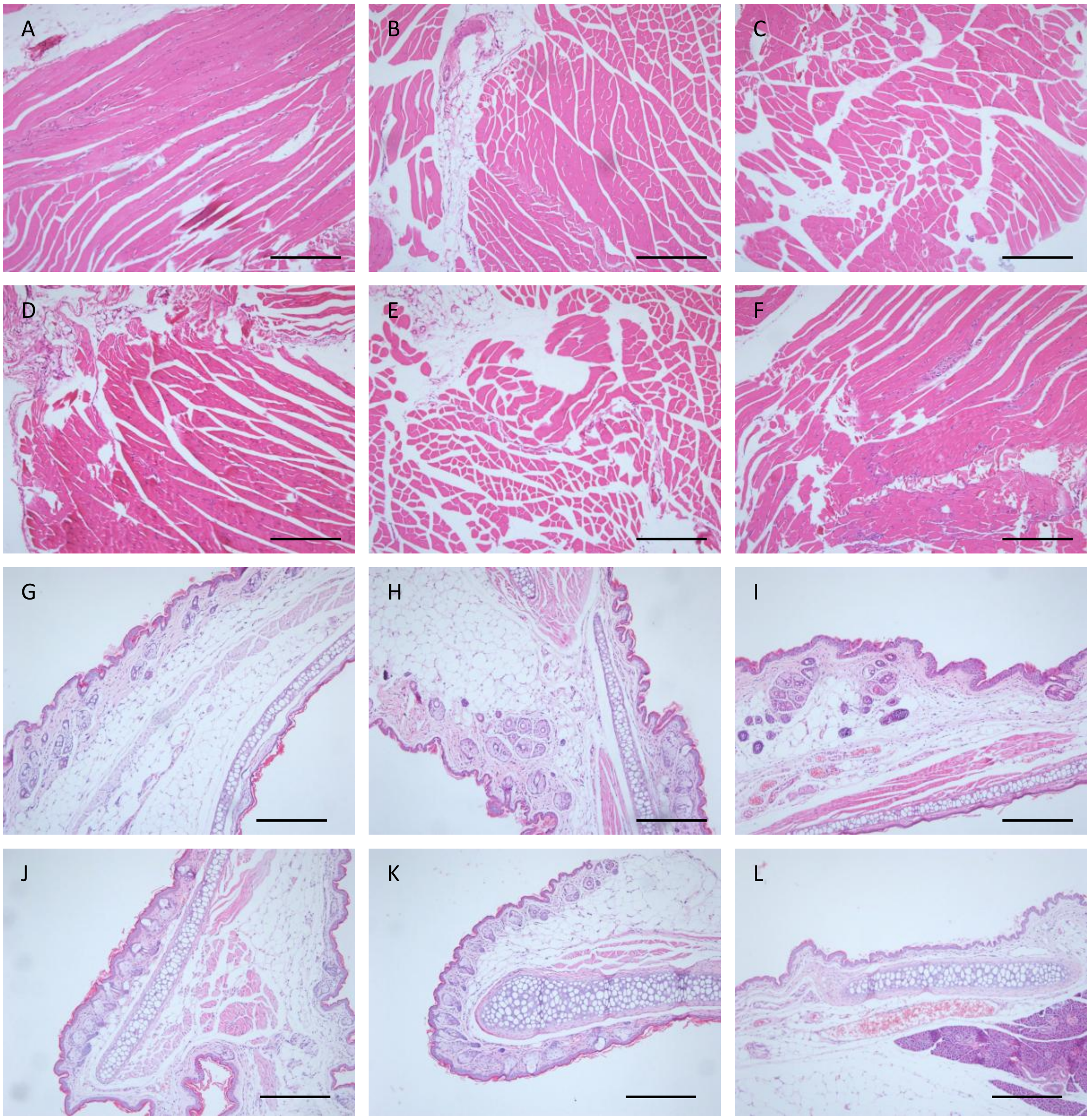

Appendix Figure 3. H&E staining of tissues in mice infected with different bacteria. Legs injected with (A) *Streptococcus* spp.; (B) *N. subflava*; (C) *Actinomyces* spp.; (D) *L. casei*; (E) *V. parvula* and (F) cocktail bacteria. Eears injected with (G) *Streptococcus* spp.; (H) *N. subflava*; (I) *Actinomyces* spp.; (J) *L. casei*; (K) *V. parvula* and (L) cocktail bacteria. Bar, 100µm.

Appendix Table 1: Distribution of bacteria at the genus level (top 10) in the lesion tissue of OFG patients.

Taxonomy	patient 1	patient 2	patient 3	patient 4	patient 5	patient 6
Neisseria	0.32%	0.24%	0.13%	0.57%	0.10%	34.34%
Enhydrobacter	0.57%	0.79%	13.88%	0.18%	0.22%	0.02%
Streptococcus	0.63%	0.81%	0.58%	0.69%	1.09%	13.60%
Pseudomonas	0.96%	1.11%	0.68%	0.73%	12.71%	0.59%
Lactobacillus	4.24%	11.04%	7.25%	5.51%	3.17%	0.59%
Delftia	3.56%	8.16%	1.02%	0.33%	0.59%	0.03%
Geobacter	0.10%	0.07%	0.24%	0.11%	7.95%	0.00%
Bacteroides	5.37%	1.00%	7.13%	3.03%	1.36%	0.15%
Rothia	0.10%	0.03%	0.25%	0.03%	0.06%	4.54%
Actinomyces	0.08%	0.31%	0.41%	0.59%	0.14%	4.54%
Others	84.07%	76.44%	68.43%	88.22%	72.62%	41.60%

Genera with ≥1% abundance on average are highlighted. The color reflects relative abundance from low (light red) to high (red).

Appendix Table 2: Occurrence frequency of organism in relation to isolates.

Organism	Frequency/ Percentage (%)	Organism	Frequency/ Percentage (%)
Streptococcus	30/100	*Bifidobacterium*	4/14.3
Neisseria	26/85.7	*Collinsella*	4/14.3
Actinomyces	21/71.4	*Dialister*	4/14.3
Haemophilus	21/71.4	*Eikenella*	4/14.3
Veillonella	21/71.4	*Prevotella*	4/14.3
Romboutsia	19/64.3	*Agathobacter*	2/7.1
Blautia	17/57.1	*Bacteroides*	2/7.1
Escherichia	17/57.1	*Capnocytophaga*	2/7.1
Faecalibacterium	17/57.1	*Gemella*	2/7.1
Lactobacillus	13/42.9	*Granulicatella*	2/7.1
Campylobacter	9/28.6	*Intestinimonas*	2/7.1
Fusobacterium	9/28.6	*Klebsiella*	2/7.1
unidentified_Lachnospiraceae	9/28.6	*Peptoclostridium*	2/7.1
Enterococcus	6/21.4	*Porphyromonas*	2/7.1
Pantoea	6/21.4	*Ruminococcus*	2/7.1
Roseburia	6/21.4		
